# Optimizing the Dielectric and Mechanical Performance of 3D-Printed Cellulose-Based Biocomposites and Bionanocomposites through Factorial Design for Electrical Insulation Application

**DOI:** 10.3390/polym16152117

**Published:** 2024-07-25

**Authors:** Morgan Lecoublet, Mohamed Ragoubi, Nathalie Leblanc, Ahmed Koubaa

**Affiliations:** 1 UniLaSalle, Unité de Recherche Transformations et Agro-Ressources (ULR 7519 UniLaSalle—Université d’Artois), 76130 Mont-Saint-Aignan, France; lecm57@uqat.ca (M.L.);; 2 de Biomatériaux, Campus de Rouyn-Noranda, Campus de Rouyn-Noranda, Université du Québec at Abitibi-Témiscamingue (UQAT), 445, boul. de l’Université, Rouyn-Noranda, QC J9X 5E4, Canada

**Keywords:** 3D printing, biocomposites, bionanocomposites, dielectric analysis, mechanical analysis, electrical insulation application, lightweight materials

## Abstract

Materials for low-permittivity and electrical insulation applications need to be re-engineered to achieve sustainable development. To address this challenge, the proposed study focused on the dielectric and mechanical optimization of 3D-printed cellulose-based composites for electrical insulation applications. Two different fillers, microcrystalline cellulose (MCC) and nanocrystalline cellulose (NCC), were used to create biocomposites and bionanocomposites, respectively, blended into a polylactic acid (PLA) matrix. The effects of infill ratio, printing temperature, and filler content on dielectric and mechanical properties were measured using an incomplete L9 (3^3) factorial design. The findings showed that the infill ratio was the most significant factor influencing the properties tested, directly attributable to the increase in material availability for polarization and mechanical performance. The second most influential factor was the filler content, increasing the polarity of the tested composites and decreasing the toughness of the biocomposites and bionanocomposites. Finally, printing temperature had no significant effect. Results for the biocomposites at a 50% infill ratio, 200 °C printing temperature, and a weight content of MCC of 15% gave a 60% higher tensile-mode stiffness than neat PLA printed under the same conditions, while exhibiting lower dielectric properties than neat PLA printed with a 100% infill ratio. These results pave the way for new lightweight materials for electrical insulation.

## 1. Introduction

Developing new materials for low-permittivity (low-k) dielectric applications is a key area of research nowadays. The worldwide electronic materials market in 2022 has been estimated at USD 65.7 billion, increasing by 6% over the next 5 years [1]. The issues of recycling, limiting fossil resources, and sustainable development require the replacement of conventional polymers such as polyethylene (PE) and propylene (PP). Biobased polymers are one of the most promising options. However, several limitations need to be considered to ensure their industrial applications. These new materials have lower dielectric performance than their synthetic counterparts. Nakatsuka in 2011 reported that polylactic acid (PLA) had a 60% higher permittivity compared to polyethylene [2]. Moreover, Hegde et al. in 2015 also noted that PLA had a higher electrical conductivity than PP [3]. To help promote these materials, it is important to either find methods to improve their electrical insulating properties or to confer them new properties, such as enhanced mechanical performance.

Three-dimensional printing is a new processing technology that is potentially very well suited to rethinking the design of products [4,5]. Unlike conventional processing technologies such as injection molding, it allows the control of the internal design of products. This technology involves the extrusion of molten plastics through a moving nozzle to produce solid parts based on computer-assisted design (CAD). This technology has shown promising results in the field of electrical insulating applications. In 2018, Barbosa et al. manufactured an electrical insulator for the development of a new generation of smart grid electrical insulators [6]. They obtained 3D-printed electrical insulators capable of withstanding an electrical tension of 35 kV, validating their use for 13.8 kV voltage applications. In 2018, Li et al. characterized the dielectric performances of 3D-printed PLA parts [7]. At 50 Hz and room temperature, PLA showed a dielectric constant (ε’) of 3.25 and an electrical conductivity (σ_DC_) of 2 × 10^−16^ S·cm^−1^. As it exhibited a dielectric strength (E_BR_) of 30 kV mm^−1^, 3D-printed PLA was acceptable for a low-voltage-level system, according to the authors. This technology can control the quantity of material inside the piece (infill ratio) as well as the printing temperature through which the material is melted (printing temperature). According to Yang et al., electrical conductivity was proportionally linked to the printing temperature [8]. An increase in printing temperature will promote interfacial adhesion of the layers, which will increase the layer bonding quality and thus promote the electron flow. Recently, Kuzmanić et al. demonstrated that a direct correlation between the dielectric constant and infill ratio for PLA specimens existed [9]. By varying the infill ratio from 100 to 33%, they reduced the dielectric constant of PLA from 3.0 to 2.2 using a square-shaped infill pattern.

Moreover, Zhang et al. showed that it is possible to adjust the dielectric constant of 3D-printed PLA parts by fine-tuning the infill ratio to produce Fresnel lenses [10]. However, reducing the infill ratio leads to reduced mechanical properties, as the literature has broadly shown [11,12,13,14]. To counterbalance this effect, it is possible to insert fillers that improve mechanical properties, such as cellulose fillers. Adding 15% of microcrystalline cellulose by weight into a PLA matrix can improve the Young’s modulus from 3.6 to 4.4 GPa [15]. However, cellulose filler has been reported in the literature to increase both the dielectric constant and conductivity of biobased polymers by adding more polar components than the matrix [16,17,18], which may limit their suitability for electrical insulation applications.

The proposed study aims to understand how the design of cellulose-based materials can influence both their dielectric and mechanical properties. The cellulose-based fillers chosen for the study are microcrystalline cellulose (MCC) and nanocrystalline cellulose (NCC) to maintain the logic of 100% compostable materials. A polylactic acid matrix was chosen due to its good availability and printability. To study the combined effect of filler content, printing temperature, and infill ratio, an incomplete L9 (3^3) factorial experimental design was chosen. This study will investigate the potential of using 100% plant-based materials for 3D printing and electrical insulation, which to our knowledge has not yet been performed.

## 2. Materials and Methods

### 2.1. Raw Materials

The used PLA was a PLI-005, supplied by Natureplast (Natureplast, Mondeville, France), with a given melt flow index (MFI) of 25–35 g·10 min^−1^ (ISO 1133 @ 190 °C). Microcrystalline and nanocrystalline cellulose (MCC and NCC, respectively) were used to reinforce the PLA. MCC was supplied by J. Rettenmaier (J. Rettenmaier, Fosston, MN, USA), obtained from bleached kraft pulp, and NCC (DextraCel Nano HP) was supplied by Anomera (Anomera, Montreal, QC, Canada).

### 2.2. Filler Silanization and Masterbatch Processing

An MCC and NCC silanization step was performed according to the protocol used by Dammak [19]. This step is very important because fiber/matrix bonding is a key aspect of the use of microcrystalline and nanocrystalline cellulose as a reinforcement material [20]. The fillers were then washed to remove the remnants of the reaction and dried at 60 °C for 24 h to remove the moisture. Infrared results confirmed the presence of Si–O–C binding on the filler surfaces, and therefore the presence of a compatibilizer (Figure S1). TGA results of the compatibilized fillers presented a thermal stability increase compared to the non-compatibilized fillers (Figure S2). The compatibilized MCC had a median and mean length of 34.1 and 51.9 ± 47.0 µm, respectively, and a median and mean width of 16.6 and 22.9 ± 19.1 µm (Figure S3). A melt-based masterbatch intermediate step was carried out for the NCC-based composites and achieved on a Haake Minilab II (Haake Minilab II, Thermo Scientific, Waltham, MA, USA). This microcompounder was operated with a batch extrusion of 5 g of PLA/NCC blend. The PLA and NCC were conditioned at 60 °C until the moisture content of the components fell below 1%. The masterbatch was prepared with a weight content of NCC (W_NCC_) = 20%, then melted with a screw speed of 70 RPM at 180 °C for 1 min. Once obtained, the extrudates were finally granulated.

### 2.3. Extrusion Processing

The extrusion step was performed by the Coalia company (Coalia, Thetford Mines, QC, Canada) with a twin-screw extruder, a temperature of 190 °C, and a screw speed between 70 and 100 RPM. Die pressure was measured between 22 and 24 bars. Table 1 shows the obtained composites. The maximal MCC and NCC content by weight was fixed at 15% and 5%, respectively, according to previous laboratory results. Once extruded, the extrudates were pelletized and oven-dried at 60 °C to remove the remaining moisture. 

### 2.4. Three-Dimensional Printing Processing

To obtain the 3D printing filaments, a 3DEVO filament maker (3DEVO, Utrecht, NLD) was used with a temperature profile of 170–185–185–180 °C, a screw speed of 3.5 RPM, and a cooling power of 40%. A CR-10 Max (CR-10 Max, Creality, Shenzhen, China) 3D printer was employed. The filaments were oven-dried at 60 °C just before printing to remove any traces of moisture. Table 2 presents the used parameters for 3D printing. These parameters were chosen as they provided the best compromise for all the different conditions. Neat PLA posed no printing problems, but the bed had to be sprayed with a lacquer to improve adhesion and prevent delamination during the printing of the high-filled biocomposites. In addition, no issues were experienced during the printing of the bionanocomposites, but the printing of the biocomposites with W_MCC_ = 15% caused some clogging and spool filament diameter stability issues.

### 2.5. Application of Factorial Design

**Figure 1:**
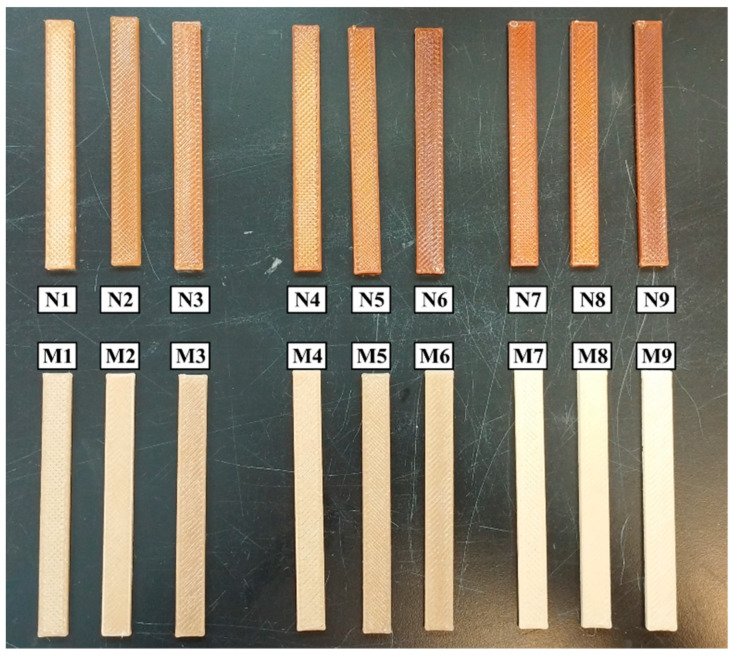
Produced conditions for the flexural tests. Flexural specimens were 120 × 12.7 mm in size.

The factorial design presented in Table 3 was employed for the conception of cellulose-based composites. The filler content (W_f_) and infill ratio were chosen as the first and second factors. The minimum infill ratio was set at 50% to avoid the collapsing phenomenon due to the rather important MFI of the used PLA. The third factor chosen was the printing temperature (T_printing_). The lowest and highest temperatures were set according to the printability range for which all composites and neat PLA could be correctly printed. These temperatures were found at 190 °C and 210 °C, respectively. Figure 1 presents the different conditions produced for flexural tests. Neat PLA specimens were added to these conditions to study the effect of infill ratio and T_printing_ on the neat matrix scale and are shown in Table 4. All samples were stored in a desiccator before being characterized.

### 2.6. Methods Used

#### 2.6.1. Broadband Dielectric Spectroscopy (BDS) Analysis

BDS analyses were carried out with a high-frequency dielectric spectrometer, the Keysight E4991A (Keysight E4991A, Agilent Technologies, Santa Clara, CA, USA). The frequency range of the used high-frequency BDS was 1 MHz to 1 GHz. Analyses for both conditions were performed on 10 × 4 mm disks, at 20 °C. A total of 5 samples per condition were tested.

#### 2.6.2. Mechanical Analysis

Tensile analyses were performed on a Zwick Z020 universal test machine (Zwick Z020, Zwick Roell Group, Ulm, Germany) according to the ASTM D638 Type 1 standard, equipped with a 20 kN load cell and with a crosshead speed of 2 mm·min^−1^. The elongation of the sample was monitored with a Zwick Roell extensometer. A total of 3 different specimens per condition were tested at room temperature.

Three-point bending flexural analyses were also performed on a Zwick Z020 universal test machine (Zwick Z020, Zwick Roell Group, Ulm, Germany) according to the ASTM D790 standard, equipped with a 20 kN load cell and a crosshead speed of 2 mm·min^−1^. To comply with the ASTM D790 standard, the gap between the two supports was set at 64 mm. A total of 3 specimens per condition were tested at room temperature. The thickness of the tensile and flexural specimens was set at 4 mm based on the equipment available in the laboratory.

#### 2.6.3. Impact Analysis

Impact tests were performed on a Zwick IZOD (Zwick IZOD, Zwick Roell Group, Ulm, Germany) with the ASTM D256 standard. For the owned test equipment, the impact arm had an initial energy of 2.75 J. A total of 10 specimens for each condition were tested at room temperature. The width of the specimens was set at 10 mm, based on the equipment available in the laboratory.

#### 2.6.4. Scanning Electron Microscopy (SEM) Analysis

SEM analysis was performed with a Jeol JSM IT200 (JSM IT200, Jeol, Akishima, Japan) to characterize the microstructure of the N5 specimen. The test was carried out on a hot-pressed, cryo-fractured, and carbon-coated specimen. The test was carried out at 5 kV with a ×200 magnification.

#### 2.6.5. Statistical Analysis and Graphical Representation

Statistical analyses were carried out by performing one-way ANOVA tests on MINITAB 17 (Pennsylvania State University, State College, PA, USA) software. Furthermore, the presented graphs were traced with Python 3.11.2 (Python Software Foundation, Fredericksburg, VA, USA) using the Matplotlib and Pandas modules.

## 3. Results and Discussions

### 3.1. Influence of Infill Ratio and Printing Temperature on 3D-Printed PLA

**Figure 2:**
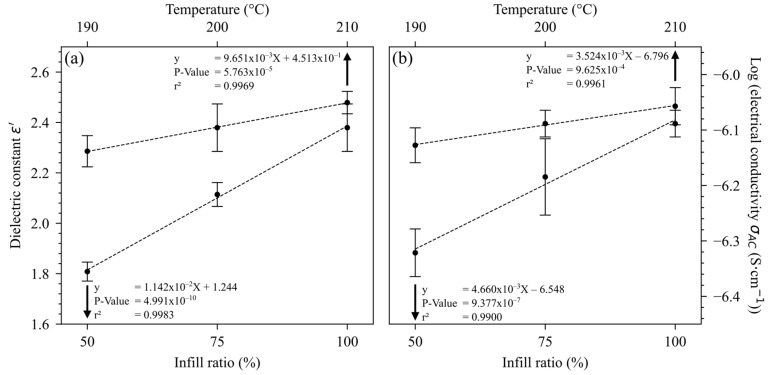
Influence of infill ratio and printing temperature on (**a**) dielectric constant ε’ and (**b**) log (electrical conductivity σ_AC_).

A preliminary study was carried out on 3D-printed PLA to examine the influence of printing temperature and infill ratio used for the factorial design. Figure 2 shows the influence of T_printing_ and the infill ratio on dielectric properties. For all conditions, the influence of T_printing_ and infill ratio was significative on the dielectric constant and electrical conductivity. The measured behaviors were linear, with an excellent correlation factor. The positive effect of the infill ratio on the dielectric constant has already been noted in the literature [9,10]. Zhang et al. also demonstrated a linear variation in the dielectric constant with the infill ratio [10]. The addition of voids through porosity decreased the polarizability potential of the material, as air has a permittivity ~1 compared to the permittivity of PLA (~3) [9,16]. In addition, the effect of printing temperature could be linked to a better printing quality, reducing defects and porosity. Concerning the influence on electrical conductivity, it has already been noted that increasing the printing temperature reduced the gap between the printed filaments and increased the interface quality [21], favoring the passage of electrons by reducing the probability of boundary area defects [8]. The increase in electrical conductivity with infill ratio was also observed by Pentek et al. [22]. It is also worth noting that for neat PLA, dielectric properties were more sensitive to the infill ratio than to temperature, even if the effect on the latter was significative.

**Figure 3:**
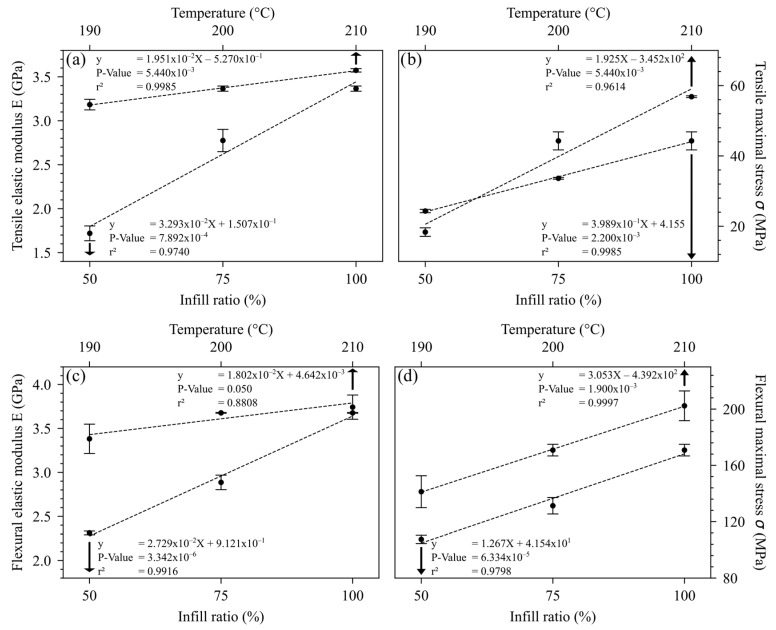
Influence of infill ratio and printing temperature on (**a**) tensile elastic modulus, (**b**) tensile maximal stress, (**c**) flexural elastic modulus, and (**d**) flexural maximal stress.

Figure 3 shows the influence of printing temperature and infill ratio on the mechanical properties of 3D-printed PLA. For all conditions, the influence of T_printing_ and infill ratio was also significative. The measured behaviors were also linear, with a good correlation factor. The modulus decrease in tensile and flexural modes with a decreasing infill ratio has been reviewed in the literature [13,23]. For maximal stress, Gunasekaran et al. also found an increase in the maximal stress in the traction and flexural mode with an increasing infill ratio [24]. According to Samykano, a lower infill ratio decreased the material’s availability to withstand internal mechanical forces, resulting in a decrease in the maximum stress [14]. Concerning the printing temperature influence, as explained above, T_printing_ played a positive role in the quality of the interface, and therefore on its ability to withstand stress.

**Figure 4:**
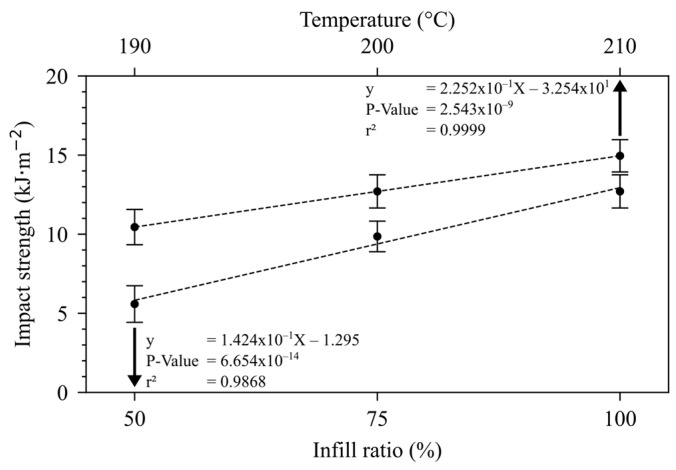
Influence of infill ratio and printing temperature on impact strength.

Figure 4 shows the influence of printing temperature and infill ratio regarding impact strength results. As impact strength represents the ability of a material to absorb energy through mechanical shocks, the noted behavior was expected, given the previous results that the infill ratio and T_printing_ had positive impacts on its ability to withstand mechanical stress. In 2022, Dharmalingam et al. also observed a near halving of the impact strength of 3D-printed PLA when the infill ratio was reduced from 100% to 50%, similar to our findings [25].

### 3.2. Dielectric Properties

**Figure 5:**
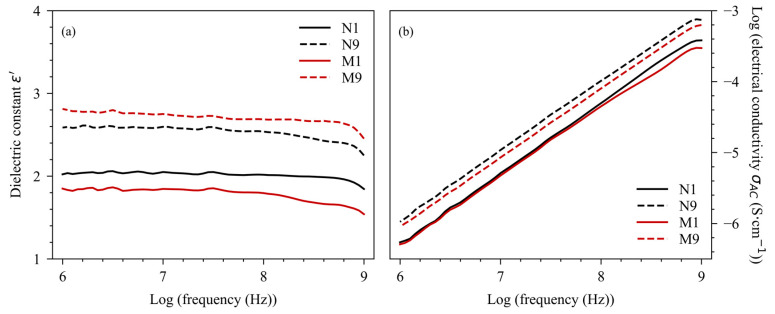
Dielectric properties versus frequency of biocomposites and bionanocomposites, (**a**) dielectric constant ε’ and (**b**) log (electrical conductivity σ_AC_).

**Figure 6:**
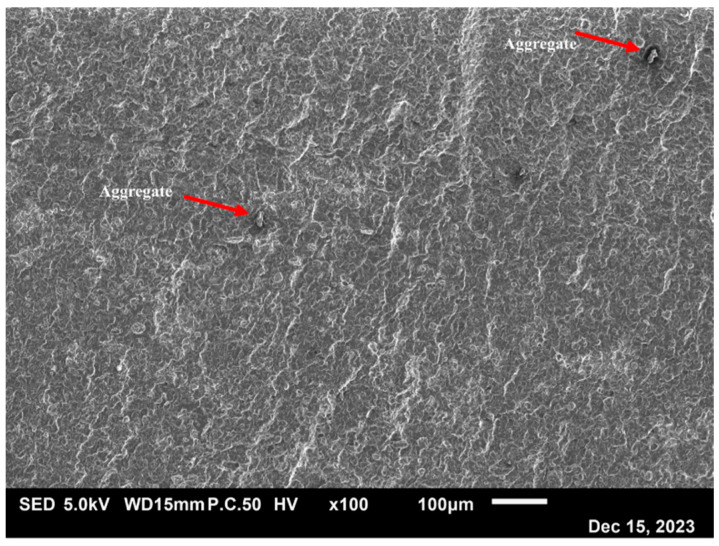
SEM picture of aggregate evidence in PLA at a W_NCC_ = 5%.

Figure 5 shows the dielectric properties obtained via BDS at room temperature from the biocomposites and bionanocomposites, using different factors (W_f_, infill ratio, and T_printing_). Measured at 1 MHz, the lowest dielectric constant was obtained for M1 at 1.85, corresponding to a W_MCC_ = 5%, an infill ratio = 50%, and a T_printing_ = 190 °C. The highest dielectric constant was obtained for M9 at 2.81, corresponding to a W_MCC_ = 15%, an infill ratio = 100%, and a T_printing_ = 210 °C. The decrease in ε’ with frequency has been widely reported in the literature [16,17,18]. A reduction in the molecular mobility of polar groups was observed when a high-frequency electric field was applied, reducing the polarization potential of the material. Under equivalent conditions and at a low filler content, the NCC-based bionanocomposites showed a dielectric constant on average 15% higher than the MCC-based biocomposites. The higher dielectric constant of the NCC-based bionanocomposites resulted from a significantly higher filler-specific surface area than that of MCC, providing more silane and hydroxyl functions on the filler surface, and therefore increasing the polarity potential of the produced composite [26]. However, at a high filler content, the biocomposite exhibited the highest dielectric constant. Preliminary results showed the presence of aggregates at 5% NCC by weight (Figure 6).

These aggregates reduced the available specific surface area of the fillers, limiting their permittivity potential. Figure 5b indicates that, measured at 1 MHz, the lowest electrical conductivity was obtained for M1 at 5.1 × 10^−7^ S·cm^−1^. The highest electrical conductivity was obtained for N9 at 1.1 × 10^−6^ S·cm^−1^, corresponding to a W_NCC_ = 5%, an infill ratio = 100%, and a T_printing_ = 210 °C. All the conditions tested showed a linear increase in electrical conductivity with frequency, which would be evidence of an electrical insulating behavior [27]. Despite the presence of the mentioned aggregates, the bionanocomposites showed greater electrical conductivity, probably due to a better diffusion of charges, which could favor the conductivity inside the material [28].

Table 5 shows the ANOVA results of the factorial design applied to the dielectric properties. The infill ratio appeared to be the most influential factor in the dielectric properties. For all conditions, the P-value was below 0.001 for both dielectric constant and electrical conductivity. These results were in agreement with preliminary findings from PLA samples. The W_f_ factor also had an overall positive impact on ε’. Badia et al. pointed out that cellulosic fillers were more polar than a PLA matrix, increasing the permittivity of the resulting composites [16]. Considering the filler type, NCC was found to exert less influence on the dielectric constant than MCC, contrary to the expected result. For an equivalent weight, NCC had a much greater specific surface area than MCC. A greater specific surface area logically provides more hydroxyl and silane groups. This reduced value could be due to aggregation phenomena at a high filler content, reducing the polarization potential. The W_f_ factor was significant for NCC but not significant for MCC regarding σ_AC_. This could be due to the charge size. It has been shown that the electrical conductivity of an insulating material can be improved if polar charges are well distributed in the matrix [28]. It can facilitate the flow of electrons induced by the phenomenon of carrier hopping, without, however, forming conductive bridges that can alter the material’s electrical insulation properties. Finally, the printing temperature had little influence on the dielectric properties. The positive effect observed for PLA specimens may be overshadowed by the overwhelming effect of the infill ratio.

### 3.3. Mechanical Properties

**Figure 7:**
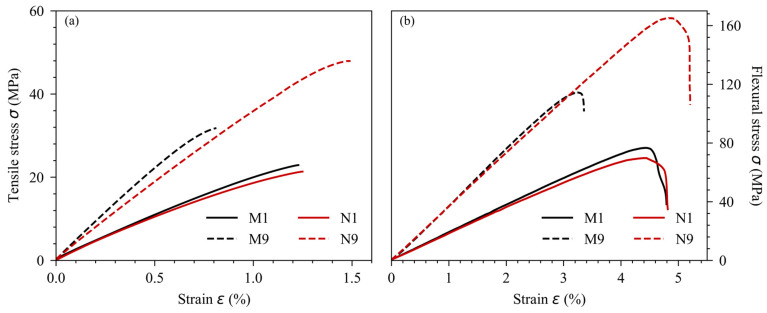
Stress-strain curves of biocomposites and bionanocomposites in (**a**) tensile mode and (**b**) 3-point bending flexural mode.

Figure 7 shows the tensile and flexural mechanical properties of the biocomposites and bionanocomposites using different factors. The same overall trends were observed for both tensile and flexural results. All conditions showed an increase in mechanical stress with the imposed elongation applied to the specimen. The tensile tests showed a brittle fracture with very little plasticization, in contrast to the flexural tests, which showed a plateau of maximal stress followed by a decrease in flexural stress, a typical behavior of three-point bending tests [11,13]. In addition, the maximum stress and elongation in the flexural tests were greater than in the tensile tests. The difference in tensile and flexural strength values could be attributed to the stress orientation in both tests: stretching in the tensile test and bending in the flexural test. At low printing temperatures, W_f_, and infill ratios, the influence of the filler nature does not seem to be significant, with a slight stiffening effect with the use of MCC. Although the addition of cellulosic fillers reduced the maximum stress of the composites compared with neat PLA, the N9s showed a much higher maximum stress than the M9s in both test modes. The presence of MCC in the PLA created microscopic stress concentration areas, resulting in a lower mechanical resistance capacity [19]. Moreover, filler size also plays a role in a material’s ability to transfer mechanical stress. In 2020, Yakubu et al. noted that smaller fillers were better dispersed in a matrix, improving the homogeneity of composites and thus increasing the maximal tensile and flexural stress [26].

Table 6 shows the ANOVA results of the factorial design applied to the mechanical properties. The infill ratio also had the greatest impact of all the factors tested, with a strong effect on the mechanical moduli and maximum stress. This positive influence is well documented in the literature. For Alafaghani et al., a higher infill ratio improved the mechanical properties by providing more materials that could undergo the applied mechanical loads [12].

Moreover, Samykano also found that a specimen with a higher infill ratio was mechanically stronger, resulting in a higher maximum stress capacity [14]. However, our infill ratio influence on elongation at maximal stress was not significant. The W_f_ also has a significant influence on the MCC-based biocomposite’s ability to withstand mechanical stress, reducing the maximal stress and elongation at maximal stress by adding defects and stress concentration areas. Printing temperature presented no significant impact. The behavior was slightly different for the NCC-based bionanocomposites. Although the effect of the infill ratio was just as noticeable as for MCC, the W_f_ had no negative effect on maximal stress and elongation. T_printing_ had a slight positive effect on the elongation at maximal stress. The results show that the reinforcing effect of cellulose fillers was minimal, probably overshadowed by the infill ratio factor. Finally, Table 7 presents the ANOVA results of the factorial design applied to impact strength. These results were very similar to the maximal stress results, as expected. According to the previous results, the effect of the infill ratio was predominant. In addition, a highly significant effect of W_f_ was noted for the MCC-based biocomposites.

## 4. Discussions

To compare the results of the produced samples with the neat PLA, Table 8 shows the dielectric and mechanical properties of PLA-0, PLA-3, M7, and N7. The latter two were chosen because they provided a relevant comparison with PLA-0 and PLA-3, as they were all printed at the same temperature.

The M7 and N7 results were globally intermediate between the 50% infill ratio PLA (PLA-0) and the 100% infill ratio PLA (PLA-3), although with a decrease in the tenacity of the composites. The lower values for the NCC-based bionanocomposites could be linked to the aggregation phenomena mentioned above, limiting the reinforcing potential of NCC. Currently, M7s could be relevant for lightweight materials, with improved stiffness compared to 50% infill PLA, while presenting a stronger electrical insulating property than neat PLA. These observations demonstrate not only the relevance of such materials, but also the need for further research to improve the mechanical properties of 3D-printed biocomposites for high-performance electrical insulation applications.

## 5. Conclusions

This study investigated the influence of infill ratio, filler content (W_f_), and printing temperature (T_printing_) on the dielectric and static mechanical properties of cellulose-based biocomposites and bionanocomposites obtained by 3D printing. An incomplete factorial design L9 (3^3) was chosen for the sample creation. A preliminary analysis of the influence of T_printing_ and infill ratio on neat 3D-printed PLA showed that such factors increased the dielectric constant, electrical conductivity, impact strength, as well as the modulus, maximal stress, and elongation at max stress in both tensile and flexural modes. Increasing the infill ratio increased the available material and therefore the associated physical quantities. The application of the factorial design showed that the infill ratio was the most influential factor of the three tested on both mechanical and dielectric properties, reducing the tested quantities as expected. The W_f_ factor had a moderate effect, generally increasing the polarity of the samples by adding polar compounds and decreasing the toughness of the biocomposites and bionanocomposites by adding stress concentration areas. The effect of temperature was negligible, indicating that this factor was marginal and potentially masked by the other factors. The produced materials were finally compared with the neat PLA. The NCC results were mediocre at a filler content of 5%, probably linked to the aggregation phenomena of nanofillers. To efficiently use NCC as a mechanical reinforcement, it may be necessary to opt for a lower filler content, such as 3%, to fully exploit the potential of this reinforcement. However, the M7 biocomposite exhibited dielectric and mechanical results for PLA with a 50% and 100% infill ratio, paving the way for biobased materials combining good electrical insulation and mechanical properties, as well as being lightweight. To go further, it would be interesting to study in depth the mechanical behavior of biocomposites by using other cellulose-based fillers such as kraft to increase the fillers’ form factor. In addition, it would also be possible to increase the filler content, as conditions with a high filler content only showed slight nozzle clogging or fluidity issues. These two perspectives would improve the material stiffness without altering too much the electrical insulating properties, validating their suitability as alternatives to conventional electrical insulation materials with equivalent mechanical rigidity, such as PE or PP.

## Figures and Tables

**Table 1:** Produced composites by extrusion.

	PLA (g)	MCC (g)	Masterbatch (g) (W_NCC_ = 20%)	W_MCC_ (%)	W_NCC_ (%)
PLA	1200	0	0	0	0
MCC-5	950	50	0	5	0
MCC-10	900	100	0	10	0
MCC-15	850	150	0	15	0
NCC-1	950	0	50	0	1
NCC-3	850	0	150	0	3
NCC-5	750	0	250	0	5

**Table 2:** Three-dimensional printing parameters.

Parameters	Values
Nozzle temperature (°C)	190–210
Nozzle diameter (mm)	0.8
Nozzle speed (mm·s^−1^)	40
Bed temperature (°C)	60
Sample thickness (mm)	3.2–4
Layer thickness (mm)	0.4
Infill ratio (%)	50–100
Infill pattern	±45°

**Table 3:** Configuration of the used factorial design.

Condition	Used Composite	Factor a W_f_ (%)	Factor b Infill Ratio (%)	Factor c T_printing_ (°C)
M1	MCC-5	5	50	190
M2	MCC-5	5	75	210
M3	MCC-5	5	100	200
M4	MCC-10	10	50	210
M5	MCC-10	10	75	200
M6	MCC-10	10	100	190
M7	MCC-15	15	50	200
M8	MCC-15	15	75	190
M9	MCC-15	15	100	210
N1	NCC-1	1	50	190
N2	NCC-1	1	75	210
N3	NCC-1	1	100	200
N4	NCC-3	3	50	210
N5	NCC-3	3	75	200
N6	NCC-3	3	100	190
N7	NCC-5	5	50	200
N8	NCC-5	5	75	190
N9	NCC-5	5	100	210

**Table 4:** Configuration of the PLA specimens.

Condition	Infill Ratio (%)	T_printing_ (°C)
PLA-0	50	200
PLA-1	75	200
PLA-2	100	190
PLA-3	100	200
PLA-4	100	210

**Table 5:** ANOVA results with F values for dielectric results.

	MCC-Based Biocomposites		NCC-Based Bionanocomposites	
	Dielectric Constant ε’	Electrical Conductivity σ_AC_	Dielectric Constant ε’	Electrical Conductivity σ_AC_
W_f_	5.2 **	2.9 n.s.	3.8 *	5.8 **
Infill ratio	46.5 ***	33.1 ***	39.9 ***	34.2 ***
T_printing_	1.8 n.s.	3.2 *	1.5 n.s.	1.6 n.s.

* Significant at 0.05; ** significant at 0.01; *** significant at 0.001; n.s., not significant.

**Table 6:** ANOVA results with F values for mechanical results.

		MCC			NCC		
		Elastic Modulus	Maximal Stress	Elongation at Max Stress	Elastic Modulus	Maximal Stress	Elongation at Max Stress
Tensile results	W_f_	0.3 n.s	7.4 **	79 ***	0.5 n.s	0.6 n.s	0.7 n.s
	Infill ratio	110 ***	9.3 ***	0.1 n.s	87 ***	140 ***	3.2 n.s
	T_printing_	0.7 n.s	1.7 n.s	0.9 n.s	0.4 n.s	0.1 n.s	0.3 n.s
Flexural results	W_f_	1.4 n.s	0.9 n.s	39.3 ***	2.5 n.s.	2.2 n.s.	2.4 n.s.
	Infill ratio	31.8 ***	24.2 ***	0.3 n.s	18.2 ***	22.1 ***	4.4 *
	T_printing_	2.4 n.s	2.3 n.s	0.3 n.s	3.1 n.s.	2.3 n.s.	3.8 *

* Significant at 0.05; ** significant at 0.01; *** significant at 0.001; n.s., not significant.

**Table 7:** ANOVA results with F values for impact strength.

	MCC	NCC
	Impact strength	Impact strength
W_f_	8.1 ***	1.3 n.s.
Infill ratio	7.7 ***	261 ***
T_printing_	2.4 n.s.	2.2 n.s.

*** significant at 0.001; n.s. not significant.

**Table 8:** Comparative study of M7 and N7 composites with 3D-printed neat PLA.

		PLA-0	PLA-3	M7	N7
Used parameters	W_f_	-	-	15% MCC	5% NCC
	Infill ratio	50	100	50	50
	T_printing_	200	200	200	200
BDS @ 1 MHz	ε’	1.81 ± 0.04	2.38 ± 0.09	2.19 ± 0.05	2.20 ± 0.03
	Log (σ_AC_ (S·cm^−1^))	–6.32 ± 0.04	–6.09 ± 0.02	–6.19 ± 0.04	–6.14 ± 0.02
Tensile	Modulus (GPa)	1.72 ± 0.08	3.37 ± 0.03	2.73 ± 0.06	1.88 ± 0.09
	Max stress (MPa)	24 ± 1	44 ± 3	19 ± 2	20 ± 1
	Elongation (%)	1.97 ± 0.08	1.45 ± 0.08	0.76 ± 0.01	1.29 ± 0.1
Flexural	Modulus (GPa)	2.31 ± 0.02	3.68 ± 0.01	2.76 ± 0.16	1.94 ± 0.06
	Max stress (MPa)	107 ± 3	171 ± 4	76 ± 3	73 ± 3
	Elongation (%)	5.34 ± 0.46	6.55 ± 0.04	3.06 ± 0.02	4.40 ± 0.33
Impact strength (kJ·m^−2^)		5.59 ± 1,16	12.7 ± 1.1	3.83 ± 1.06	6.63 ± 0.99

## Data Availability

The raw data supporting the conclusions of this article will be made available by the authors on request.

## Supplementary Materials

The following supporting information can be downloaded at: https://www.mdpi.com/article/10.3390/polym16152117/s1, Figure S1: FTIR spectra of neat and silanized MCC and NCC; Figure S2: TGA analysis of neat and silanized MCC and NCC; Figure S3: Granulometry of silanized MCC.
