# Supplementary material for: Optimizing the Dielectric and Mechanical Performance of 3D-Printed Cellulose-Based Biocomposites and Bionanocomposites through Factorial Design for Electrical Insulation Application
Source: Polymers (Basel). 2024 Jul 25;16(15):2117. doi: 10.3390/polym16152117 (PMC11314442; doi:10.3390/polym16152117)
Supplement: Supplementary file 1 [file polymers-16-02117-s001.zip › polymers-3124384-SI.pdf]

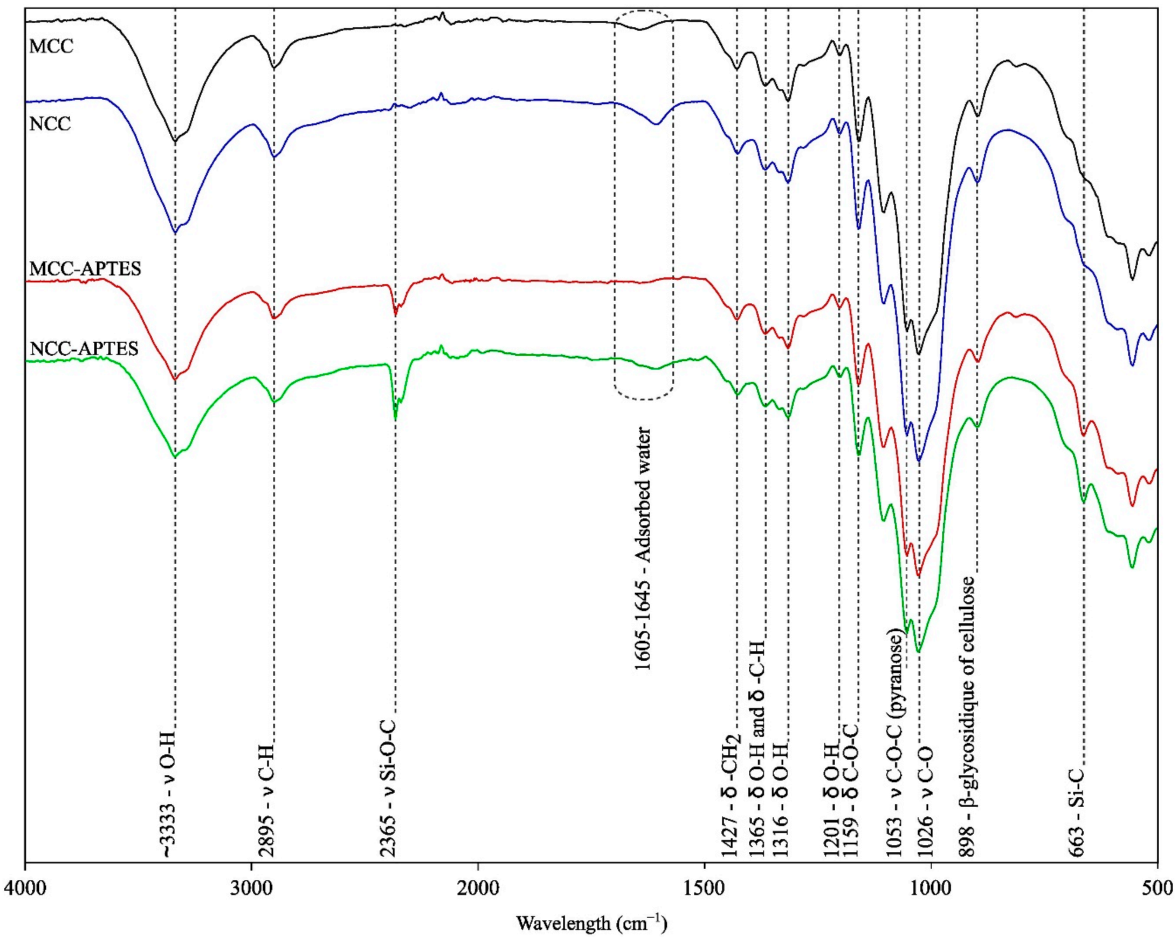

Figure S1: FTIR spectra of neat and silanized MCC and NCC

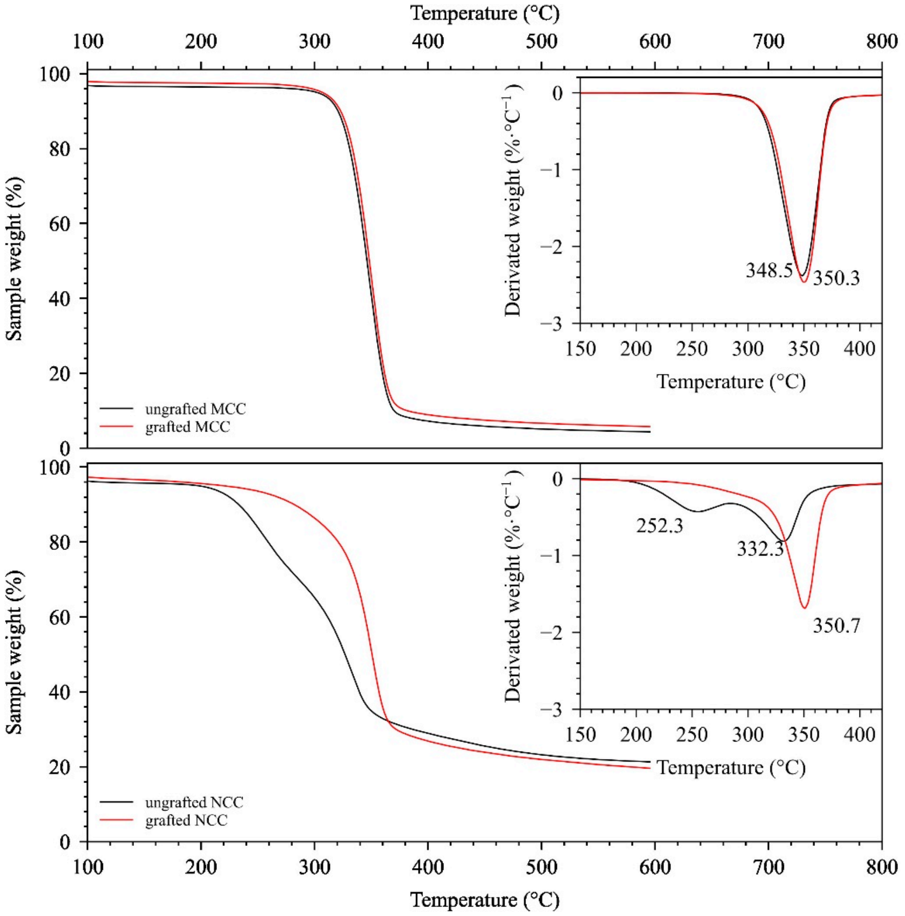

Figure S2: TGA analysis of neat and silanized MCC and NCC

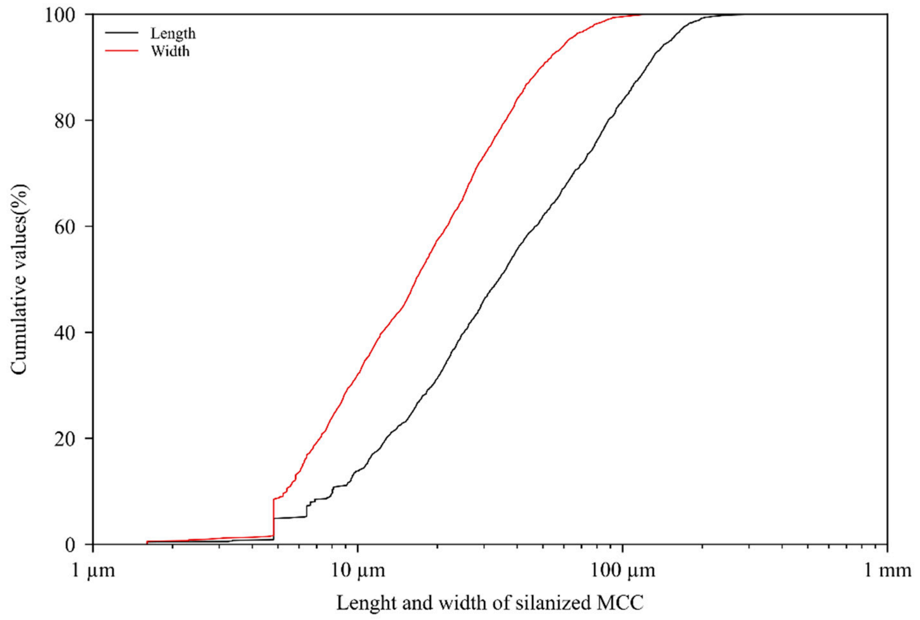

Figure S3: Granulometry of silanized MCC
